# Evaluation of Mutagenicity of Mebudipine, a New Calcium Channel Blocker

**Published:** 2010

**Authors:** Saeid Gholami, Fatemeh Soleimani, Farshad Hoseini Shirazi, Maryam Touhidpour, Massoud Mahmoudian

**Affiliations:** a *Faculty of Medicine, Iran University of Medical Sciences, Tehran, Iran.*; b * School of Pharmacy, Shahid Beheshti University of Medical Sciences, Tehran, Iran.*; c *School of Pharmacy, Islamic Azad University, Tehran, Iran.*

**Keywords:** Mebudipine, Calcium channel blocker, Salmonella TA102, Mutagenicity, S-9 mix

## Abstract

Mebudipine is a new dihydropyridine calcium channel blocker, synthesized in our laboratory, for treatment of hypertension. It has shown a better efficacy than other drugs in this group. For assessing the risks of this drug, certain safety tests in the preclinical stage have been performed. In this study mutagenic effect of mebudipine was evaluated using Ames assay that could assess the mutagenicity of drugs and their metabolites using liver enzymes (S-9 mix). This procedure is approved as a predictive test, with a high predictive value. *Salmonella *TA102 (Ames assay) was used with and without S-9 in this study. For preparing S-9 mix, rat liver enzymes induced by phenobarbital were separated in KCl 0.154 M (0.154 M), as the solvent. Mebudipine was dissolved in polyethylenglycol 400. Mutagenicity test was performed in 6 doses from 39 μg to 1250 μg per every plate, in the presence and absence of the S-9 mix. The positive control sodium azide was dissolved in a dose of 5 μg/plate dissolved in polyethylenglycol 400 and negative control was polyethylenglycol 400 with no added agent. The colony counts of all doses in plates with S-9 were between 200-400 and in plates without S9 was between100-300. The colony counts in both states (with and without S-9) of all doses were in the range suggested by Ames assay for the safe drugs and were different from the positive control groups and equal to the negative controls. Mebudipine and its metabolites were not found to be mutagen on *Salmonella *TA102, based on Ames assay.

## Introduction

**Figure1:**
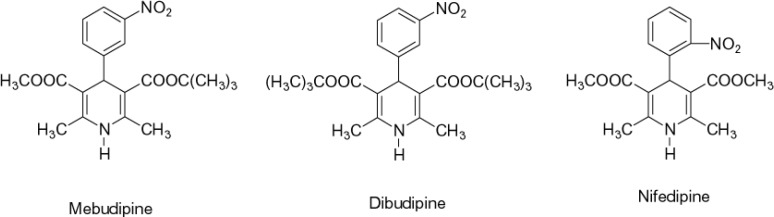
Chemical structure of old (nifedpine) and new (mebudipine and dibudipine) dihydropyridines

Calcium channel blockers have a significant role in the treatment of several cardiovascular and non-cardiovascular disorders (1, 2). Currently, extensive research is being carried out on the synthesis of new compounds of this class. Synthesis of compounds with greater tissue selectivity, longer duration of action and slower rate of absorption is the main aim of the current efforts. Such improvements in the properties of new drugs will ultimately lead to fewer side effects and improved patient compliance. In our previous study (3), it was shown that mebudipine and dibudipine, two new 1,4-dihydropyridine derivatives (Figure 1) synthesized in our laboratory, were potent relaxants of vascular and ileal smooth muscles. Inhibition of calcium-induced contraction of ileal smooth muscle (3) and reduction of calcium spikes of F1 neuronal soma membrane in Helix aspersa (4) have confirmed its calcium channel blocking properties. Relaxing The KCl-induced contractions of human internal mammary artery confirmed that the results obtained in animal studies (3) could be reproduced in human vascular preparations (5). It has been shown that these compounds have a longer half life (6) and are more active than nifedipine (7) and show a higher vascular selectivity (8). 

 It has been shown that a procedure called the Ames test is a safe way to assess mutagenicity of various agents. The Ames test is used worldwide as an initial screen to determine the mutagenic potential of new chemicals and drugs. The test is also used for submission of data to regulatory agencies for registration or acceptance of many chemicals, including drugs and biocides. The Ames *Salmonella*/microsome mutagenicity assay (*Salmonella *test; Ames test) is a short-term bacterial reverse mutation assay, specifically designed to detect a wide range of chemical substances that can produce genetic damage and leads to gene mutations. The test employs several histidine dependent *Salmonella *strains, each carrying different mutations in various genes in the histidine operon. These mutations act as hot spots for mutagens that cause DNA damage via different mechanisms. When the *Salmonella *tester strains are grown on a minimal media agar plate containing a trace of histidine, only those bacteria that revert to histidine independence are able to form colonies. The number of spontaneously induced revertant colonies per plate is relatively constant. However, when a mutagenic agent is added to the plate, the number of revertant colonies per plate increases, usually in a dose-related manner (9).

In this study, mutagenic effect of mebudipine was assessed using the Ames assay.

## Experimental

**Materials**

*Chemicals*

 Phenobarbital was made by Tolid-daru Co, Tehran, Iran. 

L-Histidine, D-biotin, sodium azide, KCL, polyethylenglycol, crystal violet and filter paper (punched and autoclaved for making sterile disk) were all purchased from the Sigma Company in USA. 

 Ampicilin, tetracycline and, mebudipine were all purchased from Pars Daroo Biopharmacy Company, Tehran, Iran. Chemical structures of mebudipine and nifedipin is shown in Figure 1. Test solutions were freshly prepared by dissolving mebudipine in polyethylenglycol 400 and kept in the dark until experimentation.

*Bactria and cultures*

 The applied strain was *Salmonella typhimurium *TA102, obtained from the toxicology department of School of Pharmacy, Shahid Beheshti University of Medical Sciences, and was frozen and stored at -73°C, to which DMSO was added as a cryoprotective agent. Salmonella TA102 has genetic characteristics, as shown in Tables 1 and 2. Cultures were prepared, based on Kristien Mortelmans and Errol Zeiger method (9). 

**Table 1:** Genotype of the *Salmonella typhimurium TA102*

**Characteristics**	**Type of genetic change**	**Results**
Histidine dependence	Mutation	No growth in the absence of histidine
Crystal violet inhibition	LPS defect (*rfa)*	No growth in the presence of crystal violet
Tetracycline resistance	Presence of plasmid pAQ1	Growth in the presence of tetracycline
Ampicillin resistance	Presence of plasmid pKM101	Growth in the presence of ampicillin

**Table 2:** Spontaneous revertant control values of Salmonella typhimurium TA102.

**With S-9 **	**Without S-9 **
200– 400	100–300

Oxoid Nutrient broth and agar were obtained from Sigma Co. in USA. 

Top agar supplemented with histidine/biotin was used to deliver the bacteria, chemicals and S-9 mix to the bottom agar. 

The formula used for the preparation of top agar was as the following: 

Distilled water .................................. 900 mL 

Agar ......................................................... 6 g 

Sodium chloride ...................................... 6 g 

Histidine/biotin solution ...(0.5 mM) 100 mL 

Top agar was prepared as follow: 

The agar and sodium chloride were added to a flask containing 900 mL of distilled water and heated for 10 min in an autoclave, in order, to melt the agar. Then, 100 mL of limited histidine and biotion solution (0.5 mM) were added to agar. The agar was stored at room temperature in the dark. When the main test was ready to undertake, the top agar was molten in boiling water. 

*Metabolic activation system S-9*

A male Sprague Dawly Rat liver was induced for microsomes by injection of phenobarbital. S- 9 mix was prepared by contusing liver (freshly separated from rat) in 0.154 M KCl at 0°C. The S9 mix was filtered through a 0.2-μm Whatman filter and stored at -73°C. Co-factors were prepared based on the procedure described by Mortelmans and Zeiger (9). 

The compounds used for making the co-factors included d-glucose-6-phosphate, nicotinamide adenine dinucleotide phosphate, potassium chloride (KCl) and dibasic sodiumphosphate (Na_2_HPO_4_.H_2_O). Sodium phosphate was purchased from Sigma Co., USA. 

**Methods**

 For preparation of the working cultures, the surface of one frozen permanent culture was scraped with a sterile inoculation loop and inoculated with 5 mL of the nutrient broth. After overnight incubation at 37°C, a loopful of the culture was streaked for purification of colonies on GM agar plates supplemented with an excess of biotin, histidine, ampicillin and tetracycline. Purification performed twice. Five single colonies from the second purification plate were picked up and transferred to a GM agar plate supplemented with the appropriate nutrient/antibiotics. This was considered as the master plate. For genetic analysis, the following steps were followed for a complete strain check (9):

- *Histidine dependence: *a loopful of the culture was streaked across a GM agar plate in the absence of histidine.
- *Histidine dependence: *a loopful of the culture was streaked across a GM agar plate

supplemented with an excess of biotin and histidine.

- *rfa marker*: a loopful of the culture was streaked across a GM agar plate supplemented with an excess of histidine. A sterile filter paper disk was placed in the center of the streak and 10 μL of a sterile 0.1% crystal violet solution was applied.
- *Presence of plasmid pKM101 (ampicillin resistance): *a loopful of the *Salmonella *was streaked across a GM agar plate supplemented with an excess of histidine and 24 μg/mL ampicillin.
- *Presence of plasmid pAQ1 (tetracycline resistance): *a loopful of a TA102 was streaked across a GM agar plate supplemented with an excess of histidine, and 2 μg/mL tetracycline.
- *Spontaneous mutant frequency: *the standard plate incorporation assay procedure was used without the inclusion of a chemical, for determining the spontaneous mutant frequency (negative control).

Bacterial growth was checked in every culture and the colonies were counted as spontaneous mutant frequency. 

After confirming the genotype, toxicity evaluation test was conducted with and without the S-9 mix. For mutagenicity determination the following procedure was adopted: 

(a) To the 13×100 mm sterile glass tubes maintained at 43°C (in water bath), the following order were added with mixing (e.g. vortexing) after each addition.

- 0.50 mL of metabolic activation (S-9) mix
- 0.05 mL of the test chemical dilution
- 0.05-0.10 mL overnight culture of the *Salmonella TA102 (*tubes were kept in a 37°C incubator for 45 min)
- 2 mL of molten top agar that thawed in 45°C (in water bath) were added at the end.

(b) The contents of the test tubes were then mixed and poured onto the surface of GM agar plates. 

(c) When the top agar set (2-3 min), the plates were inverted and placed at 37°C for 48 h. 

(d) The colonies were then counted and the results expressed as the number of revertant colonies per plate. 

Sodium azide was included as the positive control, with a dose of 5 μg/plate. 

Stages were carried out in yellow light, for prevention of UV radiation on bacteria. All steps followed the Mortelmans and Zeiger method (9). 

## Results

This investigantion showed that mebudipine is not a mutagenic agent for *Salmonella *TA102. The colony counts of all doses and positive and negative controls could be seen in Table 3. 

## Discussion

The Aim of this study was evaluation of the mutagenic effect of mebudipine using the Ames assay which is known as a highly predictive test (9). As suggested by Zeiger et al., it is better not to use statistical analysis for the Ames procedure. The range of colony counts should be considered and compared with the suggested range for safe drugs (between 100-300 colonies without S9 and 200-400 colonies with S9) (9). Based on the results obtained, the colony counts of all plates in all doses were in the range suggested for non-mutagenic agents in both states, with and without S-9 mix (Table 3) (9). Mebudipine was not found to be mutagenic on *Salmonella *TA102, with and without S-9. 

**Table 3:** The colony counts observed after 48 h

**Test compound**	**Dose/Plate**	**Mutant colonies per plates (mean ± SD) **	
		**With S9**	**Without S9**
Polyethylenglycol400 (negative control)	50 μL	260 ± 24	214 ± 37
Sodium azide (positive control)	5 μg/plate	> 3000	> 3000
Mebudipine	39 μg/plate	235 ± 13	198 ± 27
	78 μg/plate	287 ± 37	136 ± 17
	156 μg/plate	225 ± 22	140 ± 26
	312 μg/plate	342 ± 57	178 ± 33
	625 μg/plate	310 ± 46	214 ± 34
	1250 μg/plate	270 ± 43	139 ± 21

 In a study carried on a new dihydropyridine type calcium channel blocker, diperdipine, by Herzog et al, no mutagenic effect was reported (10). In another study by Obaseki et al., nifedipine did not show any mutagenic effects (11). There is no report on the carcinogenic effects of calcium channel blockers, but some association has been found between CCBs and risk of cancer (12, 13). This could be due to the blocking of calcium channels, consequently disturbing apoptosis in cells. 

For definite confirmation of the safety of mebudipine in terms of its mutagenicity, a confirmed positive control for metabolic activation system such as 2-aminoantheracene and 2-aminoflourene should have been involved in our study. An investigation has to be performed in the presence of these agents to confirm the property of the metabolic activating system. For better contrast, solvents that are suggested by Zeiger et al., like DMSO and ethanol, could be better (9) for use, since when the colonies are counted, a similarity between the color of the solvent, polyethyleneglycol, and the colonies could create some problems in counting the colonies. The reason is that, the background does not have a good contrast and hence, it would be difficult to see the colonies. 

In conclusion, it could be said that mebudipine and its metabolites are not mutagenic on *Salmonella *TA102, but as suggested by Mortelmans and Zeiger, a test with two different strains have to be performed to confirm that mebudipine is not definitely mutagenic in the *Salmonella *Ames assay (9).
